# Anxiety, emotional processing and depression in people with multiple sclerosis

**DOI:** 10.1186/s12883-017-0803-8

**Published:** 2017-02-23

**Authors:** Marie-Claire Gay, Catherine Bungener, Sarah Thomas, Pierre Vrignaud, Peter W Thomas, Roger Baker, Sébastien Montel, Olivier Heinzlef, Caroline Papeix, Rana Assouad, Michèle Montreuil

**Affiliations:** 1 Psychology Department, University of Paris West, Nanterre, France; 2 Laboratory of Psychopathology, University of Paris Descartes, Paris, France; 3 Health Psychology, Université Paris Sorbonne Cité, Paris, France; 4 Clinical Research Unit, Faculty of Health and Social Sciences, Bournemouth University, Bournemouth, UK; 5 Psychology Department, University of Paris 8, St Denis, France; 6 Neurology Department, Hospital of Poissy-St-Germain en Laye, Paris, France; 7 Neurology Department, GHPS Pitié Salpêtrière, Paris, France

**Keywords:** Multiple sclerosis, Anxiety, Depression, Emotional Processing, Coping, Mood, Predictors

## Abstract

**Background:**

Despite the high comorbidity of anxiety and depression in people with multiple sclerosis (MS), little is known about their inter-relationships. Both involve emotional perturbations and the way in which emotions are processed is likely central to both. The aim of the current study was to explore relationships between the domains of mood, emotional processing and coping and to analyse how anxiety affects coping, emotional processing, emotional balance and depression in people with MS.

**Methods:**

A cross-sectional questionnaire study involving 189 people with MS with a confirmed diagnosis of MS recruited from three French hospitals. Study participants completed a battery of questionnaires encompassing the following domains: i. anxiety and depression (Hospital Anxiety and Depression Scale (HADS)); ii. emotional processing (Emotional Processing Scale (EPS-25)); iii. positive and negative emotions (Positive and Negative Emotionality Scale (EPN-31)); iv. alexithymia (Bermond-Vorst Alexithymia Questionnaire) and v. coping (Coping with Health Injuries and Problems-Neuro (CHIP-Neuro) questionnaire. Relationships between these domains were explored using path analysis.

**Results:**

Anxiety was a strong predictor of depression, in both a direct and indirect way, and our model explained 48% of the variance of depression. Gender and functional status (measured by the Expanded Disability Status Scale) played a modest role. Non-depressed people with MS reported high levels of negative emotions and low levels of positive emotions. Anxiety also had an indirect impact on depression via one of the subscales of the Emotional Processing Scale (“Unregulated Emotion”) and via negative emotions (EPN-31).

**Conclusions:**

This research confirms that anxiety is a vulnerability factor for depression via both direct and indirect pathways. Anxiety symptoms should therefore be assessed systematically and treated in order to lessen the likelihood of depression symptoms.

## Background

Multiple sclerosis (MS) is an autoimmune degenerative disease of the central nervous system associated with significant behavioural and emotional sequelae [1–3]. People with MS have to contend on a daily basis with a range of physical, cognitive and psychological symptoms, such as walking and mobility limitations, pain, fatigue, depression, memory and concentration difficulties [4]. MS impacts on all spheres of people’s lives including employment, relationships and social life, leisure and activities of daily living [5]. People with MS face considerable unpredictability and uncertainty due to the variable nature of the disease course; for example, risks of relapses, hospital admissions and of further disability developing [6]. As there is currently no cure for MS and it is typically diagnosed in the prime of life, people live with these challenging symptoms and variable disease course over many years [5].

It is, therefore, not surprising that anxiety disorders have been reported in the literature as being present in between 36 – 54% of the MS population with approximately 30% of people with MS experiencing symptoms consistent with generalised anxiety disorder [7–9]. More than half of people with MS experience depression at some point during the course of their illness [10, 11]. Alexithymia, a difficulty in identifying and describing emotions, is also common; affecting up to 42% of people with MS. [12, 13] Research on coping (the way people manage their relationships with their environment to adjust to their disease) [14] suggests that symptoms are worsened by an emotion-centred approach [15, 16].

However, despite the high comorbidity of anxiety and depression in people with MS, little is known about the relationship between them. Brown et al. [17] showed that anxiety and depression predict each other with anxiety predicting later depression. Gay et al. [18] indicated that anxiety is a strong predictor of depression and that its impact on depression is heightened by the presence of alexithymia and a lack of social support.

Both anxiety and depression involve emotional disruptions or perturbations. Emotions are very rapid adaptive responses consisting of physiological, cognitive and behavioural elements [19]. These are felt by the individual in terms of positive or negative emotions.

According to Watson and Clark’s model of anxiety and depression [20, 21], depression is characterised by an abnormally high level of negative emotions and an abnormally low level of positive emotions, while anxiety is linked to a high level of negative emotions but without perturbations of positive emotions. Emotional balance refers to the respective levels of positive and negative emotions experienced by an individual. There is an emotional imbalance in anxiety and depression disorders [22, 23].

Emotional perturbations can also occur as processes. Baker et al. have developed a model of emotional regulation [24, 25] that draws upon Rachman's conceptualisation of emotional processing [26]. Rachman proposes that certain behavioural signs (e.g. intrusive and repetitive emotional memories) indicate that distressing emotional events have not been properly ‘emotionally processed’ or ‘absorbed’ [26]. Difficulties can occur at different stages of emotional processing in terms of registration, appraisal, experience, awareness and expression. Five emotional dysregulation processes have been identified [25]: “suppression” referring to an excessive control of emotions; “signs of unprocessed emotion”, reflecting cognitive and behavioural signs of incomplete processing; “unregulated emotion”, consisting of an inability to control one's emotions; “avoidance”, referring to the avoidance of negative emotions and “impoverished emotional experience” consisting of a detached experience of emotions due to poor emotional insight.

A key issue is how emotional perturbations (imbalances in positive and negative emotions and emotional processing deficits) lead to depression and whether anxiety exerts an impact directly on depression in MS or whether it influences depression via factors related to emotional processing. The aim of the current study was to explore the relationship between anxiety and depression and the relevance of emotional processing, emotional balance, and coping to depressive symptomatology.

## Methods

All the required ethical authorisations were obtained from the French Ethics Committee (Comité de Protection des Personnes (CPP)) and all study participants provided written informed consent.

### Participants and procedure

A cross-sectional questionnaire study was undertaken. Participants were patients recruited from the day care unit of the neurological departments in three French university hospitals (CHU Metz, CHI Poissy-Saint Germain, CHU La Pitié-Salpêtrière) with a neurologist-confirmed diagnosis of MS (revised McDonald criteria) [27]. Recruitment took place in 2013.

The research study was described to potential participants by the neurologist. If they wished to participate, a psychology Masters student, trained for the study, obtained their written informed consent and then collected socio-demographic and medical information from them. Participants completed the battery of self-reported outcome measures in the day care unit. The trainee was available to answer questions if required.

### Descriptors

#### Socio-demographic and clinical variables

Socio-demographic variables (age, gender, education level and marital status) were obtained by the psychology student and clinical and disease-specific variables (type of MS and time since diagnosis) were obtained from medical notes.

#### Physical Disability

The Expanded Disability Status Scale [28] was used as a measure of physical disability. The Expanded Disability Status Scale (EDSS) was administered by an experienced neurologist and provided a measure of functional status. The EDSS is divided into eight functioning systems (pyramidal, cerebellar, brainstem, cerebral/mental, bowel and bladder, visual function, sensory, and other). Impairment in each system is graded separately by means of neurological examination. EDSS scores range as steps from 0 – 10 in 0.5 increments. Levels 1.0 – 4.5 refer to people with a high degree of ambulatory ability and the subsequent levels 5.0 – 9.5 refer to a loss of ambulatory ability. The range of main categories include (0) = normal neurologic exam*;* to (5) = ambulatory without aid or rest for 200 m, disability severe enough to impair full daily activities*;* to (10) = death due to MS.

#### Outcomes

All outcomes were self-reported.

#### Anxiety and depression

The Hospital Anxiety and Depression Scale (HADS) is a 14-item scale for use as a brief instrument for detecting the intensity of depression and anxiety in patient populations [29]. The HADS has few somatic items so is unlikely to confound depression with physical symptoms such as pain and fatigue and has been validated for use in the MS population [30]. Scores for the depression and anxiety subscales can range from 0 – 21 respectively, with a score >10 indicating probable anxiety or depression. The French adaptation of the HADS confirmed Zigmond and Snaith’s [29] original two factor structure and has been shown to possess good psychometric properties [31]. Internal reliability was 0.79 for the anxiety subscale and from 0.82 for the depression subscale. The correlation between the two subscales was significant but moderate (*r* = .47), representing 22% of the common variance [31].

#### Emotional processing

The Emotional Processing Scale (EPS-25) is a 25-item self-report questionnaire designed to identify and measure emotional processing styles and potential deficits in healthy individuals and those with psychological or physical disorders [24, 25]. It comprises five subscales, each with five items that are rated on a 10-point (0–9) attitudinal scale: suppression (excessive control of emotional experience and expression), signs of unprocessed emotion (intrusive and persistent emotional experiences), unregulated emotion (inability to control one's emotions), avoidance (avoidance of negative emotional triggers), impoverished emotional experience (detached experience of emotions due to poor emotional insight). A higher score indicates poorer emotional processing with a possible mean score range of 0–9. In the original English language version of the EPS developed in the UK [25] these five factors explained 59.4% of the total variance and overall internal reliability (Cronbach's Alpha) was high (α = 0.92), ranging from 0.70 – 0.80 for the five respective factors. The EPS-25 has been translated into numerous languages and norms have been produced for a wide range of clinical and non-clinical populations. A French version has been developed (Gay et al., not yet published). In the French adaptation the five factors explained 61.5% of the total variance and overall internal reliability (Cronbach’s Alpha) was 0.91 and ranged from 0.68 – 0.84 for the five respective subscales. A French sample of healthy adults from the general population (*N* = 75) had a mean (SD) total EPS score of 2.5 (1.04) and mean (SD) scores for the respective subscales, as follows: Suppression = 3.0 (1.89); Signs of Unprocessed Emotion = 3.3 (1.79); Unregulated Emotion = 1.9 (1.31); Avoidance = 2.9 (1.36); Impoverished Emotional Experience = 1.4 (1.19). French data from 349 people with MS showed significant differences on every subscale compared to healthy adults with the exception of the Suppression subscale. The mean (SD) total EPS score for the MS sample was 3.2 (1.69) and means (SDs) for the respective subscales were: Suppression = 3.5 (2.39); Signs of Unprocessed Emotion = 3.7 (2.80); Unregulated Emotion = 2.6 (1.86); Avoidance = 3.5 (1.90) and Emotional Experience = 3.5 (1.89).

#### Emotional balance

The Positive and Negative Emotionality Scale (EPN-31) [22, 23] measures emotionality; in particular, the self-reported frequency with which 31 emotional states have been experienced in the past month. It consists of 31 items and produces three main scores: a positive emotionality score, a negative emotionality and a surprise emotionality score. The answer format is a 7-point scale, ranging from 1 “Not experienced at all” to 7 “Experienced this affect several times each day”. For the French adaptation these three factors explained 58.2% of the total variance and internal reliability was good with Cronbach’s Alphas between 0.80 and 0.95 for all main scores and between 0.72 and 0.90 for the six subscores (joy, tenderness, anger, fear, sadness, shame) [23]. A reference population comprising 948 French healthy adults (mean (SD) age = 41.4 (9.64) years) had a mean (SD) negative emotion score of 32.0 (14.30) and a mean positive emotion score of 70.1 (16.00) [23].

#### Alexithymia

The Bermond-Vorst Alexithymia Questionnaire [32, 33] (BVAQ) is a 40-item self-report measure which comprises two parallel versions, each with 20 items. We have used the B form. Five factors are assessed: difficulty in verbalising feelings, difficulty in identifying feelings, lack of emotional excitability, externally-oriented thinking, poor fantasy life. Each item is rated on a five-point Likert-type scale, ranging from 1 (strongly agree) to 5 (strongly disagree), with a maximum score of 100. A score over 52 is indicative of the presence of alexithymia. The French version of the BVAQ has demonstrated good psychometric properties with Cronbach’s Alpha coefficient 0.83 for a sample of 322 Belgian students [33].

#### Coping

The Coping with Health Injuries and Problems-Neuro (CHIP-Neuro) Questionnaire consists of 24 items assessing the coping strategies of people with neurological conditions [34, 35]. The questionnaire includes six different coping strategies for neurological health problems: emotional regulation (seven items), seeking well-being (five items), active distraction (three items), information seeking (three items), palliative coping (three items) and cognitive avoidance (three items). Items are rated on a numerical scale ranging from 0 – “not at all” to 5 – “a lot”, with a possible total score ranging from 0 – 120. A dominant coping strategy can be identified according to respondents’ highest subscale score. The CHIP-Neuro has been validated with a French sample of people with multiple sclerosis and Parkinson’s disease (*N* = 307) with 48% of the variance explained by the six factor solution. The internal reliability was good with Cronbach’s Alphas ranging between 0.80 and 0.82 for the six subscales.

### Statistical analysis

The variables introduced in the analysis were checked for normality by examining histograms and considering values for kurtosis and skewness. Descriptive statistics were used to summarise these data and Pearson’s Product Moment Correlation Coefficients were used to explore associations between the various domains of interest.

Path analysis was used to estimate the strength of the direct and indirect relationships between the variables of interest. Chi-squared, the Root Mean Square Error of Approximation (RMSEA) and the Comparative Fit Index (CFI) statistics were used as measures of fit.

SPSS Version 21 for Windows was used to undertake the descriptive and correlational analyses and AMOS 20.0 structural equation modeling software for the path analyses. We imputed missing data using a multiple imputation method based on a Monte-Carlo Markov Chain algorithm [36]. The chosen algorithm imputes values for each case by drawing, at random, from the conditional distribution of the missing values given the observed values, with the unknown model parameters set equal to their maximum likelihood estimates.

## Results

In total 189 participants completed the questionnaire battery. The mean age was 47.2 years (SD = 12.50; range = 18–78). Females (females = 121; males = 68) comprised approximately two-thirds of the sample reflecting gender prevalence ratios in the general multiple sclerosis population. Just over half the participants were married or in long term relationships and just over two-thirds had received education up to the age of 17 years. In terms of type of MS, 107 (57%) of the sample had relapsing remitting MS, 54 (29%) secondary progressive MS and 28 (15%) primary progressive MS. The Expanded Disability Status Scale (EDSS) was used as an index of functional status and the sample had a mean (SD) EDSS score of 4.7 (2.37) and a mean [(SD) range] disease duration of 15.0 [(9.29) 2–18] years.

Levels of missing total score data for the outcome measure questionnaires were very low (less than 3%) with the exception of the EPN-31 scale for which 8% of total scores were missing.

Descriptives for the self-reported outcome variables for the entire sample are presented in Table 1. Overall the sample had a self-reported level of depression in the normal range (HADS depression score M = 8.0, SD = 4.19). However, 19% of participants scored between 8 and 10 on the HADS depression subscale indicating possible depression and 28% scored over 10 indicating probable symptoms of depression. Using a cut-off score >10 on each respective subscale for probable anxiety and/or depression, 16/189 (8.5%) participants had both anxiety and depression; 8/189 (4.2%) had anxiety only; 37/189 (19.6%) had depression only and 128/189 (67.7%) had neither depression nor anxiety. There was a statistically significant difference (t(187) = 3.26, *p* < .001)) between HADS depression scores for females (M = 8.7, SD = 4.37) and males (M = 6.7, SD = 3.50) with females tending to score higher than males. Self-reported levels of anxiety were in the normal range (M = 5.8, SD = 3.78). However, 17% had scores between 8 and 10 suggesting possible anxiety and 13% had scores >10 indicating probable anxiety.

**Table 1:** Descriptive statistics for the self-reported outcomes

	Descriptives
	Unless otherwise specified [(mean (SD) range)]
Outcome measure	Entire sample (*N* = 189)
Hospital Anxiety and Depression Scale (HADS) *(higher scores, more distress)*	
Depression subscale (HADS-D)	8.0 (4.18) 1–19
Score <8 [N (%)]	100 (52.91%)
Score 8–10 [N (%)]	36 (19.04%)
Score >10 [N (%)]	53 (28.04%)
Anxiety subscale (HADS-A)	5.8 (3.9) 0–18
Score <8 [N (%)]	133 (70.37%)
Score 8–10 [N (%)]	32 (16.93%)
Score >10 [N (%)]	24 (12.70%)
Emotional Processing Scale (EPS-25) *(higher scores, poorer emotional processing)*	
Suppression	4.0 (2.64) 0–9.0
Signs of Unprocessed Emotions	4.1 (2.35) 0–8.4
Unregulated Emotion	2.6 (1.97) 0–8.8
Avoidance	3.9 (1.99) 0–8.2
Impoverished Emotional Experience	2.6 (1.89) 0–8.2
Total EPS Score	4.3 (2.12) 0–9.0
Bermond-Vorst Alexithymia Questionnaire (BVAQ) *(higher scores, greater levels of alexithymia)*	
Difficulty in verbalising feelings	11.8 (4.09) 4–20
Poor fantasy life	11.2 (4.33) 4–20
Difficulty identifying feelings	10.9 (4.04) 4–20
Lack of emotional excitability	10.8 (4.31) 4–20
Externally-oriented thinking	10.7 (4.45) 4–20
Total BVAQ Score	55.4 (14.15) 20–95
Coping with Health Injuries and Problems – Neuro (CHIP-Neuro) *(higher scores, greater use of coping style)*	
Emotional regulation	22.0 (6.65) 7–35
Seeking Well-being	19.2 (3.18) 9–25
Active Distraction	9.1 (3.16) 3–15
Information Seeking	9.8 (4.00) 3–15
Palliative Coping	7.9 (2.99) 3–15
Cognitive Avoidance	8.3 (2.93) 3–15
Positive and Negative Emotionality (EPN-31) *(higher scores, greater emotionality)*	
Positive	47.2 (11.78) 10–70
Negative	51.0 (19.63) 18–111
Surprise	8.3 (3.98) 3–19

With the exception of the Avoidance subscale, mean EPS-25 scores were comparable to those found in the general population [26]. Thirty-six percent of the current MS sample scored more than one standard deviation above the mean on the Avoidance subscale. Females (M = 4.1; SD = 1.95) had significantly higher scores than males (M = 3.4; SD = 1.98) on the Unregulated Processing subscale, t(187) = 2.35, *p* = .02.

On the CHIP-Neuro questionnaire mean scores for the Emotion Regulation and Well-being subscales were twice as high as those for the other subscales suggesting these were the most common coping strategies used by respondents.

The cut-off for clinical alexithymia on the BVAQ is ≥53. The mean (SD) BVAQ total score for the current MS sample was 55.4 (14.15) with just over half (53%) the sample scoring at or above this cut-off.

The results showed a perturbation of emotional balance assessed by the EPN-31, with the sample self-reporting a much higher level of negative emotions (M = 51.0; SD = 19.64) and lower level of positive emotions (M = 47.2; SD = 11.79) compared to a healthy French adult reference population (*N* = 948) [30]. There was a statistically significant difference (t(187) = 2.28, *p* = .02) between negative emotion scores for females (M = 53.4, SD = 20.02) and males (M = 46.7, SD = 18.31) with females tending to score higher than males.

### The relationships between anxiety, depression, emotions, emotional processing, and functional status

The relationships between depression and the other variables were explored using Pearson’s Product Moment Correlation Coefficients (see Tables 2, 3, 4 and 5). HADS anxiety scores were moderately positively correlated with HADS depression scores (r_p_ = .41, *p* < .001). HADS anxiety scores were moderately positively correlated with total EPS scores (r_p_ = .40, *p* < .001) and moderately positively correlated with negative emotion scores on the EPN-31 (r_p_ = .35, *p* < .001). HADS anxiety scores were moderately negatively correlated with positive emotion scores (r_p_ = −.64, *p* < .001). Regarding coping, a moderate negative correlation was found with the active distraction subscale score of the CHIP-Neuro (r_p_ = -.49, *p* < .001) as well as a moderate positive association with emotional regulation-based coping (r_p_ = .36, *p* < .001).

**Table 2:** Correlations between HADS depression scores and EDSS, HADS anxiety, EPN-31 and EPS scores (*N* = 189)

	EDSS	HADS Anxiety	EPN-31 Negative	EPN-31 Positive	EPS Suppression	EPS Unprocessed	EPS Unregulated	EPS Avoidance	EPS Impoverished	EPS Total
HADS depression	-.19*	.42***	.62***	-.07 ns	.38***	.56***	.53***	.39***	.39**	.58***

* *p* <.05; ** *p* <.01; *** *p* <.001; ns Not significant

**Table 3:** Correlations between HADS depression scores and BVAQ scores (*N* = 189)

	BVAQ Verbalisation	BVAQ Fantasy	BVAQ Identification	BVAQ Excitability	BVAQ Externality	BVAQ Total
HADS depression	.03 ns	.09 ns	-.01 ns	.09 ns	.04 ns	.07 ns

* *p* <.05; ** *p* <.01; *** *p* <.001; ns Not significant

**Table 4:** Correlations between HADS anxiety scores, EPN-31 and EPS scores (*N* = 189)

	EPN-31 Negative	EPN-31 Positive	EPS Suppression	EPS Unprocessed	EPS Unregulated	EPS Avoidance	EPS Impoverished	EPS Total
HADS anxiety	.36***	-.64***	.36***	.34***	.35***	.31***	.34***	.58***

* *p* <.05; ** *p* <.01; *** *p* <.001; ns Not significant

**Table 5:** Correlation matrix for potential variables for path model

	1	2	3	4	5	6	7	8	9	10	11	12	13	14	15	16	17	18	19	20	21	22
1. Depression (HADS)	-																					
2. EDSS	-.19*	-																				
3. Anxiety (HADS)	.42***	ns	-																			
4. Suppression (EPS)	.38***	ns	.36***	-																		
5. Unprocessed (EPS)	.56***	ns	.34***	.53***	-																	
6. Unregulated (EPS)	.53***	ns	.35***	.40***	.60***	-																
7. Avoidance (EPS)	.39***	ns	.31***	.52***	.53***	.48***	-															
8. Impoverished (EPS)	.39***	ns	.34**	.52**	.53**	.48***	.55***	-														
9. EPS Total	.58***	ns	.58***	.79***	.83***	.74***	.77***	.78***	-													
10. Emotional regulation (CHIP-Neuro)	.47***	ns	.37***	.36***	.32***	.47***	.31***	.45***	.45***	-												
11. Seeking well-being (CHIP-Neuro)	ns	ns	-.26***	-.17**	ns	ns	ns	ns	ns	-.21**	-											
12. Active distraction (CHIP-Neuro)		ns	-.50***	-.27***	ns	ns	ns	-.17*	ns	ns	-.25**	-										
13. Information seeking (CHIP-Neuro)	.19**	ss	ns	ns	.16*	ns	ns	ns	ns	.27***	.20**	.34***	-									
14. Palliative Coping (CHIP-Neuro)	ns	ns	ns	ns	.16*	ns	.20**	.17*	ns	.26***	.26**	.16*	.16*	-								
15. Cognitive Avoidance (CHIP-Neuro)	ns	-.21**	-.22**	ns	ns	ns	ns	ns	ns	ns	.16*	.17*	.17*	ns	-							
16. BVAQ total	ns	ns	.18*	.20**	ns	ns	ns	ns	ns	ns	-.16*	ns	ns	ns	ns	-						
17. Verbalisation (BVAQ)	ns	ns	ns	ns	ns	ns	ns	ns	ns	ns	ns	ns	ns	ns	ns	.53***	-					
18. Fantasy (BVAQ)	ns	ns	ns	ns	ns	ns	ns	ns	ns	ns	ns	ns	ns	ns	.18*	.53***	ns	-				
19. Identification (BVAQ)	ns	ns	ns		ns	ns	ns	ns	ns	ns	-.15*	ns	ns	ns	ns	.76***	.30**	.30***	-			
20. Excitability (BVAQ)	ns	ns	ns	.19*	ns	ns	ns	ns	ns	ns	-.15*	ns	ns	ns	ns	.71***	.16**	ns	.44**	-		
21. Externality (BVAQ)	ns	ns	.16*	.20**	ns	ns	ns	.15*	.15*	ns	-.16*	ns	ns	ns	.80***	.26**	.24**	.24**	-.61*	-.15*	-	
22. Positive Emotions (EPN-31)	ns	ns	-.64***	-.20**	ns	-.17*	-.20**	-.21**	-.21**	ns	.23**	.19*	ns	ns	-.21**	ns	ns	-.16*	-.15**	-.20**	ns	-
23. Negative Emotions (EPN-31)	.62***	ns	.36***	..30***	.42***	57***	.38***	.50***	.50***	.47**	-.15*	ns	ns	ns	ns	ns	ns	ns	ns	ns	ns	ns

* *p* <.05; ** *p* <.01; *** *p* <.001; ns Not significant

Scores on the HADS depression subscale correlated positively and strongly with negative emotion scores on the EPN-31 (r_p_ = .60, *p* < .001) and with EPS total scores (r_p_ = .57, *p* < .001) and more specifically with the Signs of Unprocessed Emotions (r_p_ = .55, *p* < .001) and Unregulated Emotion subscales (r_p_ = .51, *p* < .001). HADS depression scores correlated positively and moderately with scores on the Emotional Regulation subscale of the CHIP-Neuro (r_p_ = .45, *p* < .001) and weakly and negatively with EDSS scores (r_p_ = −.18, *p* < .05).

There were no statistically significant differences between depressed and non-depressed participants in terms of alexithymia scores, F(2,186) = 1.36, *p* = .26, nor between anxious and non-anxious participants, F(2,186) = 1.99), *p* = .14.

### The study of the direct and indirect effects of the variables using path analysis model

The path analysis aimed to explore the direct and indirect effects of anxiety on depression. Functional status was retained in the model to control for MS severity. A covariance relationship between functional status and anxiety was introduced into the model to take into account the existence of a link between these variables. Variables were introduced into the model if they were significantly correlated with anxiety and/or depression (see Tables 2, 3, 4). Alexithymia was therefore not included in the model. Among the variables representing emotional processing, only “Unregulated Emotion” was selected because its correlation was one of the highest with depression. Despite a high correlation with depression, “Signs of Unprocessed Emotion” was not introduced into the model because its shared variance with “Unregulated Emotion” led to a suppressing effect on this variable. As no specific hypothesis was available on the direction of a causal link between “negative emotions” and “Unregulated Emotion”, their relationship was estimated as a covariance.

The path analysis model included both direct and indirect effects on the dependent variable “depression”. One variable (functional status) had only direct effects. One (anxiety) had direct and indirect effects mediated by “Unregulated Emotion” and by “negative emotions”. The model consists of 13 parameters. The sample size (*N* = 189) seems sufficient for a reliable estimation of these parameters at .80 power according to the *R* ^2^ value (.46) and the direct and indirect effect sizes following results from Thoemmes et al. [37].

**Fig. 1:**
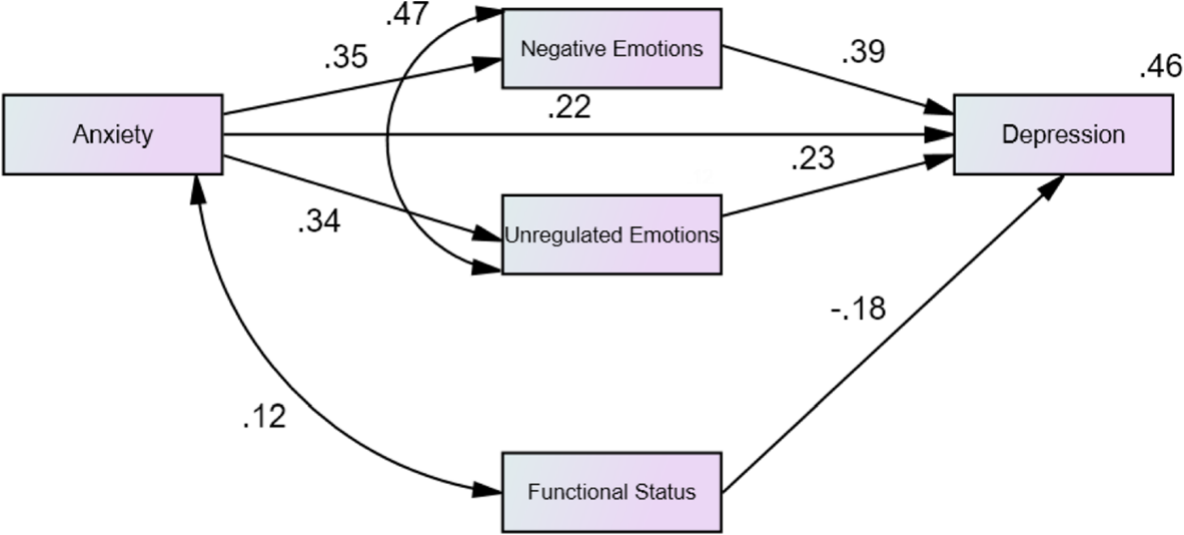
Path analysis model (*N* = 189). Standardised values, all coefficients presented are significant at *p* < .001)

The non-significant Chi-squared test statistic indicated acceptable goodness-of-fit of the model (*χ* ^2^ (2) = 4.12, *p* = .13). The Root Mean Square Error of Approximation (RMSEA) was 0.075 indicating an acceptable fit (RMSEA values < 0.08 indicate an acceptable fit), the Comparative Fit Index (CFI) was 0.98 indicating an acceptable fit (values >0.90 indicate an acceptable fit) [38]. Figure 1 presents the standardised values of the regression coefficients. All the values are significant at *p* = .001.

The model explained almost half of the variance of depression (*R* ^2^ = .46). The direct effect of anxiety on depression is estimated at .24, the indirect effects (negative emotions, Unregulated Emotion) at .22.

## Discussion

In this research our aim was to explore the relationship between anxiety and depression in a sample of people with MS and the relevance of emotional processing to depressive symptomatology. We also examined the respective contributions of several sociodemographic, clinical and psychological factors to depressive symptomatology.

In line with the literature [10, 11], the results confirmed that depression is prevalent in people with MS. HADS anxiety scores >10 were obtained by 8% of the sample which is lower than is commonly reported in the MS literature [7–9] though 17% had scores suggesting possible anxiety.

The results also indicated that people with MS had perturbations in their emotional balance, according to Watson and Clark’s model [20, 21]; they reported fewer positive emotions than the general population and more negative ones. Their mean scores were similar to those observed in people with anxiety and depressive disorders [22] which is in line with previous research in MS [15].

However, in the current study people with MS with probable anxiety disorder also showed perturbation of their positive emotions. Another difference from Watson and Clark’s model is that even non-depressed people with MS reported high levels of negative emotions and low levels of positive emotions. This result suggests that experiencing negative emotions is not sufficient to develop depression or to feel depressed. Negative emotions were nevertheless strongly implicated in depression, through dysfunctional emotional processing and anxiety.

Anxiety was a strong predictor of depression via both direct and indirect pathways. Indirect pathways were via one of the subscales of the Emotional Processing Scale, “Unregulated Emotion” and through negative emotions (EPN-31).

Functional status had an independent impact on depression. This impact was low, which is in line with the literature [18]. Anxiety and functional status were independent. The separation between anxiety and functional status indicated that functional status did not impact on anxiety. It may be that individuals’ illness representations [39] and the anticipation of possible future consequences of MS [6] may provoke anxiety above and beyond that arising from current symptoms and functional status. In turn, this anxiety may inhibit healthy emotional processing.

Coping strategies did not appear to mediate the relationship between anxiety and emotional processing nor that between anxiety and depression. While alexithymia did not contribute to the final model we cannot exclude the possibility that this was due to the specific scale we used. Future research should also include other measures of alexithymia such as the Toronto Alexithymia Scale (TAS-20) [33].

In conclusion, our model explained 46% of the variance of depression. It is consistent with Watson and Clark’s model of depression [21]; having high levels of negative emotions and few positive ones is a feature of depression. Depressed people with MS had such a configuration of emotions. The unpredictable and variable nature of MS may explain how anxiety can lead to perturbations in emotional balance.

Anxiety is a vulnerability factor for depression since it directly and indirectly induces negative emotions which can lead to depressive symptoms. A similar pattern of relationships between anxiety and depression has been demonstrated by researchers using other questionnaires specifically assessing anxiety (the State and Trait Anxiety Inventory- STAI [40]) and depression (the ZUNG Self-Rating Depression Scale [41]).

### Study limitations

It could be argued that it might have been better to assess depression and anxiety using two separate scales. However, it is challenging to assess depression in people with MS since symptoms of MS overlap with depression (asthenia, fatigue, loss of energy, psychomotor impairment, appetite disorders, sleep disorders, sexual disorders, and cognitive disorders) and an advantage of the HADS depression subscale is that it contains few somatic items, has been validated in French and has been widely used with people with MS.

The study is based on self-reported measures and we know that people with depression tend to report more negatively in retrospective recall. Additionally, it would have been good to collect information on disease modifying treatments and to have included fatigue [17] and social support in the model.

There are inherent and well-recognised limitations in drawing conclusions about directionality in cross-sectional research of this kind. Prospectively designed studies would overcome such limitations.

Despite these acknowledged limitations, our research has strengths in showing how anxiety may affect depression in people with MS. The model we have proposed explained just under half the variance of depression and so clearly there are a range of other potential contributors (such as neurological dysfunction). Nevertheless, our findings suggest that anxiety plays an important role in the presence of depression.

## Conclusion

This research confirmed that in the current sample of people with MS anxiety was a strong predictor of depression via both direct and indirect pathways. The model obtained suggested that anxiety may affect depression through unregulated emotion and negative emotions and highlights an important potential role for early intervention. We suggest several possible treatment approaches could be applied to target anxiety. Information provision for people with MS seems to increase disease-related knowledge, with less clear results on decision making and quality of life [42]. Cognitive Behavioural Therapy (CBT) has been shown to be effective for anxiety disorders in the general population [43] and should be considered for those with MS with signs of anxiety or anxiety disorders. Interventions should address both individual and social factors that support resilience such as promoting positive thinking and planning and engagement in meaningful activities. Positive psychological approaches that focus on eliciting positive emotions may provide a means of reducing or even neutralising the impact of aversive events on emotional experiences [44–46]. Emotion-focused or experiential therapies may be particularly helpful for people with poor awareness of their emotions and psychological functioning [47, 48]. Recognising the limits of the current research, there is a need for a more complete consideration of demographic, disease specific and psychosocial factors involved in the development of depression and of their respective contributions. National MS registers are ideally placed for the longitudinal exploration of these more complex path models [7].

## Abbreviations

BVAQ: Bermond-Vorst Alexithymia Questionnaire
CHIP-Neuro: Coping with Health Injuries and Problems-Neuro
EDSS: Expanded Disability Status Scale
EPN-31: Positive and Negative Emotionality
EPS-25: Emotional Processing Scale
HADS: Hospital Anxiety and Depression Scale
MS: Multiple Sclerosis
pwMS: People with multiple sclerosis
SEM: Structural equation modelling
STAI: State and Trait Anxiety Inventory
